# School absence policy and healthcare use: a difference-in-difference cohort analysis

**DOI:** 10.1093/fampra/cmae042

**Published:** 2024-09-06

**Authors:** Kirsti Wahlberg, Kristine Pape, Bjarne Austad, Andreas Asheim, Kjartan S Anthun, Johan H Bjørngaard, Gunnhild Å Vie

**Affiliations:** Department of Public Health and Nursing, NTNU Norwegian University of Science and Technology, Post box 8905, 7491 Trondheim, Norway; Department of Public Health and Nursing, NTNU Norwegian University of Science and Technology, Post box 8905, 7491 Trondheim, Norway; Trondheim Municipality, Post box 2300 Torgarden, 7004 Trondheim, Norway; General Practice Research Unit, Department of Public Health and Nursing, NTNU Norwegian University of Science and Technology, Post box 8905, 7491 Trondheim, Norway; Center for Health Care Improvement, St. Olav’s University Hospital, Post box 3250 Torgarden, 7006 Trondheim, Norway; Department of Mathematical Sciences, NTNU Norwegian University of Science and Technology, 7491 Trondheim, Norway; Department of Public Health and Nursing, NTNU Norwegian University of Science and Technology, Post box 8905, 7491 Trondheim, Norway; Department of Health Research, SINTEF Digital, Post box 4760 Torgarden, 7465 Trondheim, Norway; Department of Public Health and Nursing, NTNU Norwegian University of Science and Technology, Post box 8905, 7491 Trondheim, Norway; Faculty of Nursing and Health Sciences, Nord University, Post box 93, 7601 Levanger, Norway; General Practice Research Unit, Department of Public Health and Nursing, NTNU Norwegian University of Science and Technology, Post box 8905, 7491 Trondheim, Norway

**Keywords:** adolescent, young adult, health services research, general practice, hospitals, public policy

## Abstract

**Background:**

A national policy in Norway demanding certificates for medical absences in upper secondary school was implemented in 2016, leading to an increase in general practitioner (GP) visits in this age group.

**Objectives:**

To assess the policy’s effect on the use of primary and specialist healthcare.

**Methods:**

A cohort study following all Norwegian youth aged 14–21 in the years 2010–2019 using a difference-in-differences approach comparing exposed cohorts expected to attend upper secondary school after the policy change in 2016 with previous unexposed cohorts. Data were collected from national registries.

**Results:**

The absence policy led to the increased number of contacts with GPs for exposed cohorts during all exposed years, with estimated incidence rate ratios (IRRs) in the range from 1.14 (95% confidence intervals [CI] 1.11–1.18) to 1.25 (95% CI 1.21–1.30). Consultations for respiratory tract infections increased during exposed years. However, there was no conclusive policy-related difference in mental health consultations with GPs. In specialist healthcare we did not find conclusive evidence of an effect of absence policy on the risk of any contact per school year, but there was a slightly increased risk of contacts with ear–nose–throat specialist services.

**Conclusions:**

We found an increase in general practice contacts attributable to the school absence policy. Apart from a possible increase in ear–nose–throat contacts, increased GP attention did not increase specialized healthcare.

**Key messages**

- The Norwegian school absence policy led to increased contact with GP offices.
- Respiratory tract infections doubled, but mental health was barely affected.
- Specialist care contacts were largely unaffected by the policy.
- Specialist care ear–nose–throat diagnoses seemed more frequent as an effect.
- Few signs signalled any lasting effect after the end of policy exposure years.

## Introduction

In 2016, the Norwegian government introduced a new policy for upper secondary school students regarding school absence, with the intention of decreasing truancy and improving school presence [1]. From the autumn semester of 2016, absence in one school subject exceeding 10% must be certified to get a passing grade. Certificates for medical absences must be issued by a medical doctor or other appropriate personnel. Due to the COVID-19 pandemic, the policy was suspended from March 2020 to the end of the school year 2021/2022 [1].

An increase in consultation rates (in general practice or out-of-hours services) and drug dispensing was found in the first autumn following implementation, most markedly for respiratory tract infections [2]. An evaluation report published in 2020 [3] also showed an increase in contacts with the general practitioner (GP) offices, and to a lesser degree an increase in contacts with out-of-hours services and private specialist outpatient services, following the implementation of the policy. The policy was deemed a success in terms of school absence, which decreased by 18 hours per student the first year after implementation, though no apparent effect on continuation or completion of upper secondary education was found [3].

The Norwegian healthcare system is mostly publicly funded [4]. Nearly all Norwegian citizens have a registered regular GP, and outside of normal working hours, a primary care out-of-hours service is available. GPs are the gatekeepers for the public specialist healthcare and can refer to a relevant specialist when needed [4]. Preventive healthcare is also implemented by youth health centres and school health services [5].

Norwegian youth are a generally healthy group, with low healthcare utilization [6] and low mortality [7]. However, there is an increasing trend in healthcare utilization [6] including consultations for mental health problems in general practice [8]. Exogenously induced increases in healthcare use, like the absence policy, might have positive or negative consequences. Unnecessary contacts might increase the workload for GPs and lead to overtreatment, as indicated by increased use of antibiotics [2]. Increased help-seeking might also increase referrals to specialist services. Whether it could also affect help-seeking behaviour beyond the years in upper secondary school has not been examined previously. On the other hand, youth might benefit from GP contacts as it enhances acquaintance with their regular GP who works from a life course perspective [9], and it may provide an opportunity to detect and intervene in mental health problems at an early stage [10].

We aimed to assess whether the use of primary or secondary healthcare services at age 14–21 differed among cohorts exposed to the absence policy compared to cohorts not exposed to this policy.

## Materials and methods

### Study design

We performed a registry-based cohort study of healthcare use comparing youth exposed to the absence limit to youth not exposed using a difference-in-difference approach [11]. Assuming parallel age trends in healthcare use for different birth cohorts, the changes pertaining to the absence policy can be disentangled from time trends [11].

### Setting

Norwegian upper secondary schooling is divided into general studies (3 years) and vocational programmes (typically 2 years of schooling and 2 years of apprenticeship). Upper secondary school is normally started in the autumn of the year a person turns 16 and completed at 18 (vocational) or 19 (general studies) [12]. The absence policy is not enforced during the apprenticeship period of vocational studies when sick leave regulations for employees apply [1].

The Norwegian healthcare system is divided into the primary healthcare, organized by the municipalities, and the secondary healthcare system, organized by regional hospital trusts [4]. We subdivide specialist healthcare into somatic (including specialized rehabilitation) and specialist mental healthcare (including specialized addiction treatment).

### Participants

Norwegian youth born 1996–2003. Participants were included in the autumn of the year they turned 14 when the observation period started (July 2010) or the date of immigration. They were excluded in the autumn of the year they turned 21, at the date of emigration or death, or at the end of the observation period (31 December 2019). See Table 1 for observation and exposure years.

**Table 1.** School years of exposure, by birth year, and exposure group.

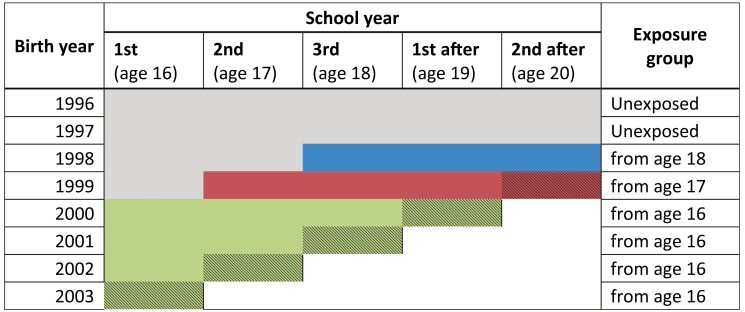

Notes: Green colour represents unexposed observed years. Red colour displays exposed years observed. Shaded school years are only observed until December 31st. In addition, all birth years were observed the 2 years before upper secondary (ninth and tenth grade).

### Data sources

Statistics Norway supplied data on population statistics, and the Control and Payment of Health Reimbursement Register (KUHR) and Norwegian Patient Register supplied information on contacts and diagnoses.

### Exposure

We defined exposure to the absence limit by belonging to a birth cohort that would be exposed to the absence limit during upper secondary, irrespective of whether the individual attended schooling (intention-to-treat analyses). We defined four categories of exposure, corresponding to the age at onset of exposure. See Table 1.

We defined school years from July 1st to June 30th the following year, named by the expected grade names. Thus 9th and 10th represent the 2 years before upper secondary, first to third represents upper secondary, while the following 2 (school) years are named «first after» and «second after».

### Outcomes

The main outcomes were (a) the number of days of contact with GP offices and (b) contact (yes/no) to a somatic specialist or specialist mental healthcare, respectively, per school year. This included simple contacts, consultations, and home visits for GP offices (chosen due to possible office-wide effects of the policy) and inpatient or outpatient contact for specialist services.

As secondary outcomes, we similarly counted the number of consultations (including home visits) with GP or out-of-hours services, respectively, and the number of GP consultations with pre-specified diagnoses per school year. We chose (1) respiratory tract infections and (2) mental health diagnoses because they are frequent among this age group and important reasons for school absence. We chose (3) acne and (4) nevus as control diagnoses, as these would not be causes of school absence (Supplementary Table 1).

We also assessed any contact with somatic or specialist mental healthcare per school year with selected diagnosis groups (Supplementary Table 1). Based on outpatient contact or discharge ICD-10 diagnosis, we chose (I) ear–nose–throat diagnoses (II) affective disorders, and (III) eating disorders to examine whether increased general practice contacts led to increasing referrals for diseases of the airways or serious mental health disorders. We initially planned to measure bipolar disorders as a more severe medical condition but included all affective disorders in the analyses because of a few cases with bipolar disease. (IV) Fractures were chosen as a control diagnosis presumably unaffected by the policy.

### Covariates

Covariates used were immigration status, parental education (both as four categories and dichotomized), and an indicator variable for inhabitants of a municipality per year. For further description see additional method description in Supplementary Materials.

### Follow-up

Follow-up started in July 2010, because in 2010 a policy was implemented that removed out-of-pocket payments for children aged 12–15 [13, 14]. The study period ended in December 2019, because of varying enforcement of the absence limit and changing healthcare use during the COVID-19 pandemic.

### Statistical analyses

We used Poisson regression, clustered by individuals, with fixed effects within municipality-year (i.e. only comparing variability within each municipality-year combination), adjusting for immigration status, parents’ highest education, and sex [15]. We measured outcomes in each age interval based on the available number of days observed as registered inhabitants.

We additionally repeated analyses in subgroups of socioeconomic status and sex and performed negative control analyses among individuals who had not started upper secondary education, as any estimated effect here would suggest bias [16]. We also repeated analyses with linear regression, to provide a measure on an absolute scale. In addition, we calculated the proportion of people in exposure groups being registered as in upper secondary schooling per school year.

We later performed two additional sensitivity analyses. One where we removed the halved school year of 2019, and another where we adjusted for half year and fixed effects for the municipality-half year.

Precision was measured with 95% confidence intervals (CI). Analyses were performed in Stata 17.

## Results

Of a total of 528 076 persons, 390 254 were from exposed birth cohorts. The study population was observed for a total of 2.8 million person years, of which 1.9 million were in ever-exposed cohorts. The overall average number of contacts to general practice per year was 2.8 days (SD 3.6). There were only minor differences in the proportion in schooling at each age comparing exposed and unexposed birth cohorts (see Supplementary Table 2).

### Primary healthcare contacts and diagnoses

**Figure 1.**
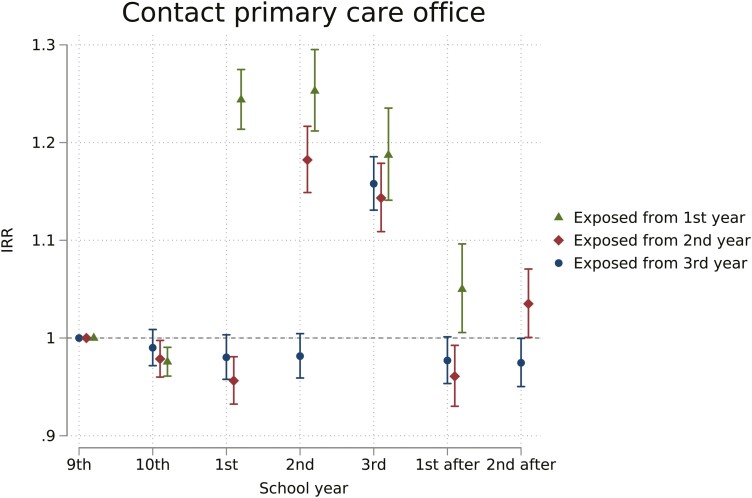
Estimated IRR of days of contact with GP offices from a difference-in-difference model. By school year and exposure groups, estimates reflect the change attributable to the school absence policy. School years were defined from July 1st to June 30th the following year and named by the corresponding grade name. Exposure groups are defined by expected age at start of exposure. Contacts include all simple contacts, consultations, and home visits not including out-of-hours services).

General practice contacts are presented in Fig. 1, comparing exposed cohorts to unexposed cohorts. For cohorts exposed from the first year of upper secondary, days of contact with GP offices were increased during first (the incidence rate ratio (IRR) 1.24, 95% CI 1.21–1.27), second (IRR 1.25, 95% CI 1.21–1.30), and third (IRR 1.19, 95% CI 1.14–1.24) school year. Cohorts exposed from the second year of upper secondary had an increase during the second (IRR 1.18, 95% CI 1.15–1.22) and third upper secondary year (IRR 1.14, 95% CI 1.11–1.18). Cohorts exposed from the third year of upper secondary had an increase during the third year (IRR 1.16, 95% CI 1.13–1.19). Before the onset of exposure, the exposed cohorts were down to 4% fewer days of contact compared to unexposed cohorts. After upper secondary years, the exposed groups ranged from 4% fewer to 5% more days of contact compared to the unexposed comparison. Linear regression models indicated that the absence policy increased days of contact with GP offices by 0.6–0.8 days per school year. Similarly, GP consultations only were 18%–30% higher comparing exposed to unexposed years (Supplementary Fig. 1).

**Figure 2.**
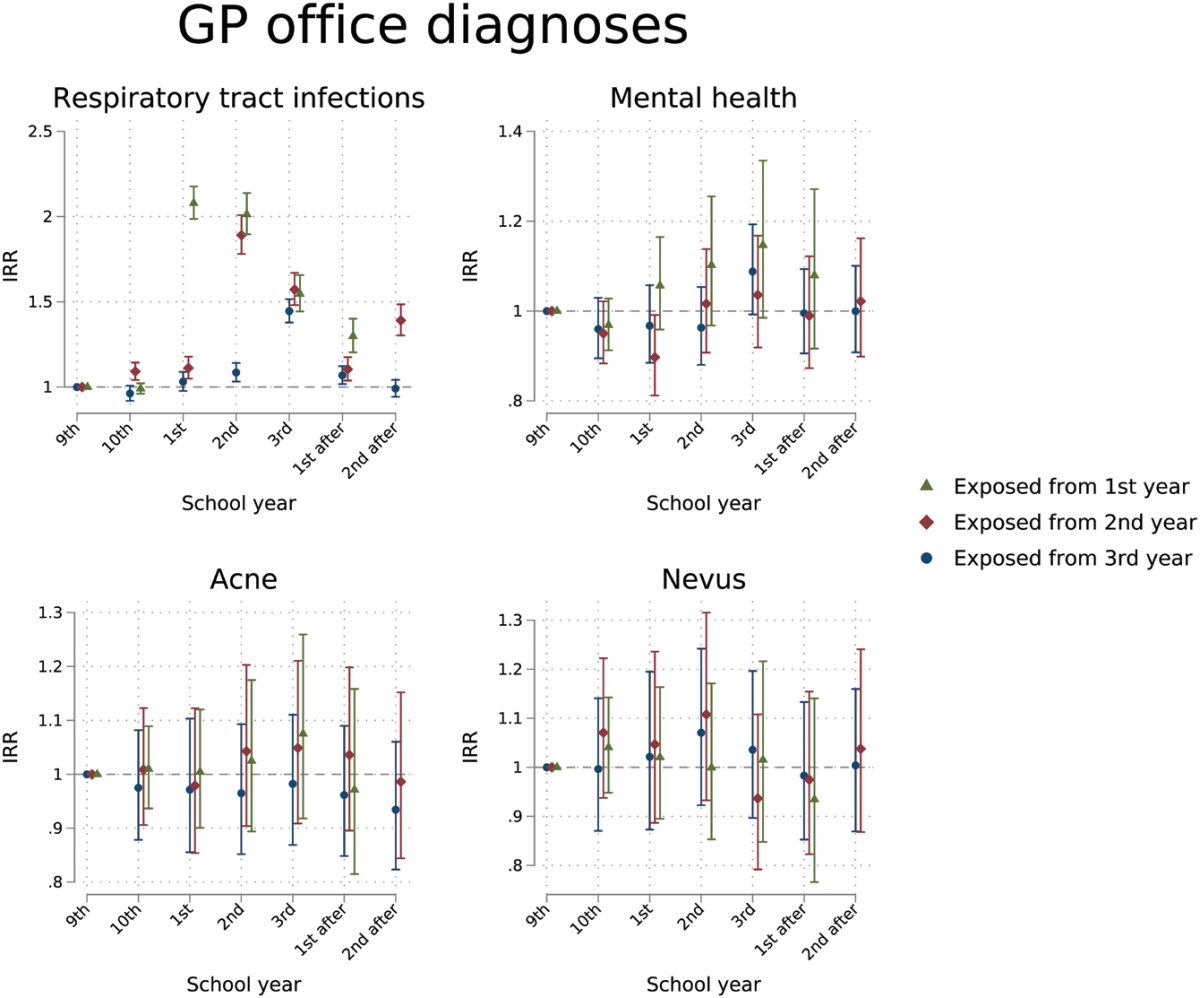
Estimated IRR of the number of consultations with selected diagnoses in a GP office per school year from a difference-in-difference model. By school year and exposure groups, estimates reflecting the change attributable to the school absence policy. School years were defined from July 1st to June 30th the following year and named by the corresponding grade name. Exposure groups are defined by expected age at start of exposure.

Figure 2 compares the frequency of GP consultations with the four given diagnosis groups among exposed cohorts to those not exposed. Exposed cohorts had an increase in consultation rates for respiratory tract infections during upper secondary years (highest the first year of upper secondary for those exposed from first year, IRR 2.08, 95% CI 1.99–2.18). After upper secondary years, the effect was minor and varied between groups. Consultation rates for mental health diagnoses, acne, and nevus had only minor changes.

We observed an increased use of out-of-hours services in some of the groups exposed to the school absence policy, but the timing did not correspond to exposure (Supplementary Fig. 2).

### Specialist healthcare contacts and diagnoses

**Figure 3.**
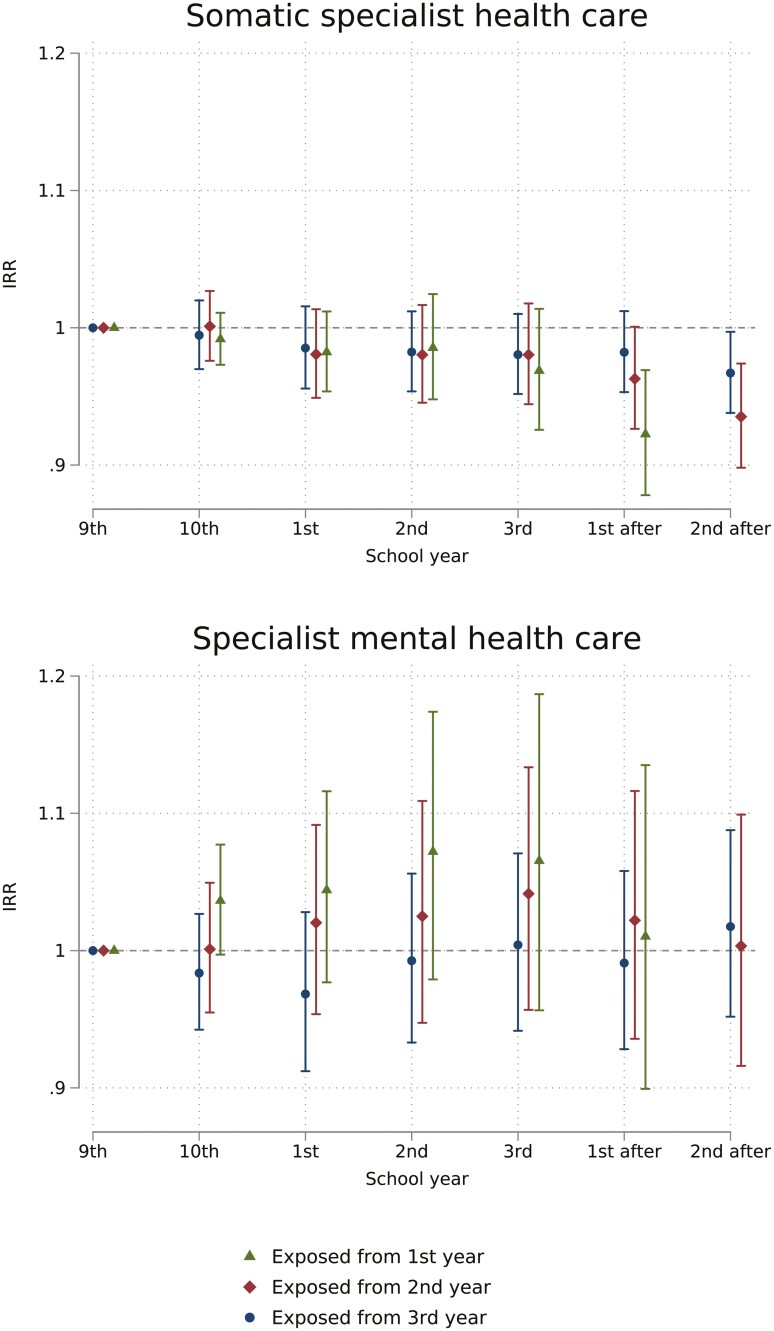
Estimated IRR of days of inpatient or outpatient contact with somatic specialist healthcare and specialist mental healthcare from a difference-in-difference model. By school year and exposure groups, estimates reflect the change attributable to the school absence policy. School years were defined from July 1st to June 30th the following year and named by the corresponding grade name. Exposure groups are defined by expected age at the start of exposure.

There was weak evidence for any change in contacts with somatic specialists or specialist mental healthcare attributable to the absence limit, see Fig. 3. Students exposed from the first year of upper secondary had a slight decrease in somatic specialist healthcare the only observed year after upper secondary school (IRR 0.92, 95% CI 0.88–0.97), while there was a slight decrease in contacts the second school year after for students exposed from second (IRR 0.94, 95% CI 0.90–0.97) and third school year (IRR 0.97, 95% CI 0.94–1.00).

**Figure 4.**
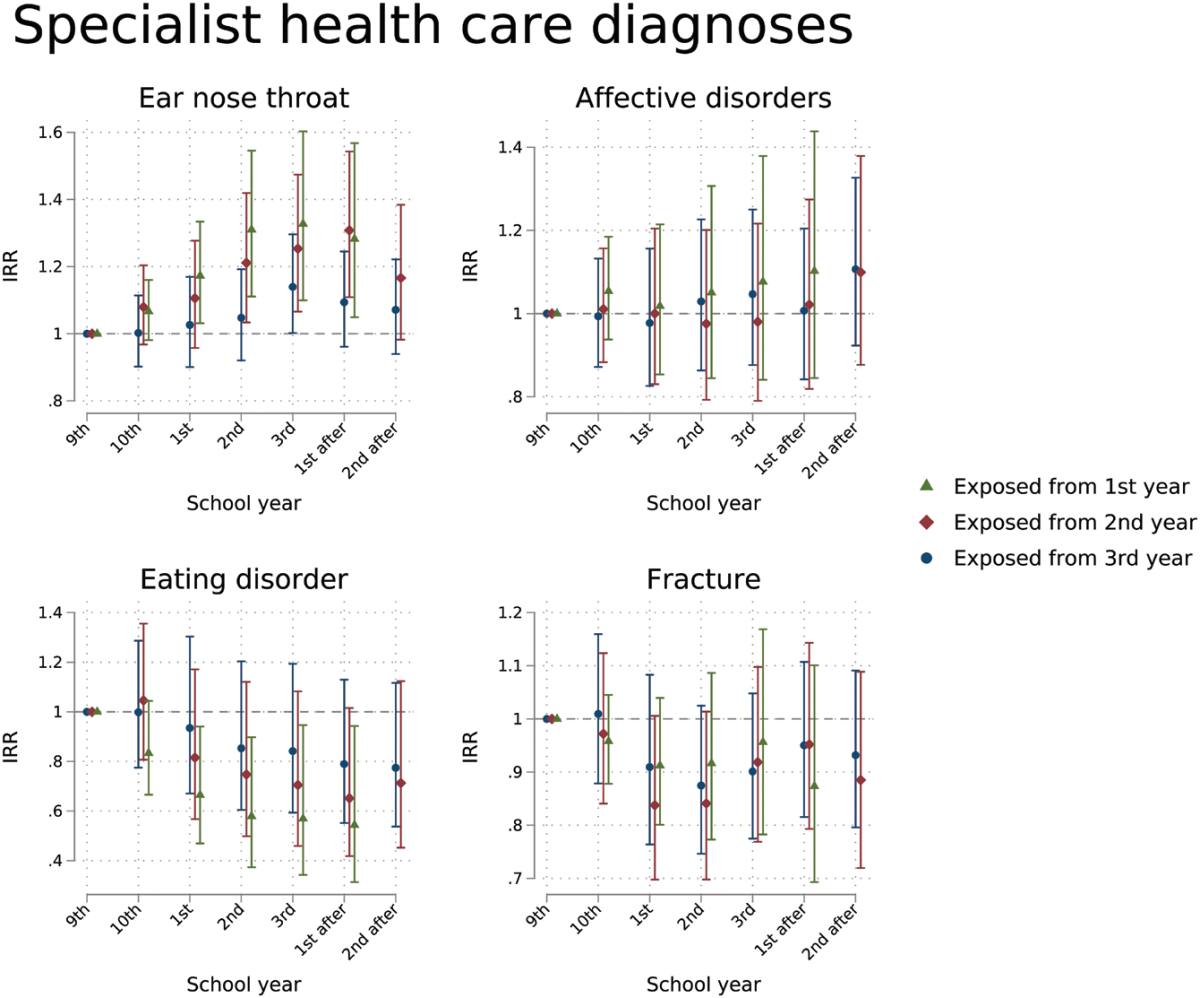
Estimated IRR of probability of contact with selected diagnoses in specialist healthcare per school year from a difference-in-difference model. By school year and exposure groups, estimates reflect the change attributable to the school absence policy. School years were defined from July 1st to June 30th the following year and named by the corresponding grade name. Exposure groups are defined by expected age at the start of exposure.

The risk of having any contact with specialist care for ear–nose–throat diagnoses was higher during exposed upper secondary years (highest in the third year for those exposed from first year, IRR 1.33, 95% CI 1.10–1.60) and temporarily after upper secondary school years for those exposed from first and second years (Fig. 4). Contacts for eating disorders decreased during upper secondary school years most markedly for those exposed from first school year, and somewhat after exposure years.

### Subgroup analyses, negative control, and additional sensitivity analyses

In separate analyses, effect estimates for primary care contacts were smaller among youth of parents with lower, compared to higher education (Supplementary Fig. 3). However, exposed cohorts seemed to have a slightly negative trend before exposure, most so among those with lower educated parents. We found only minor differences in absolute changes between educational groups (Supplementary Fig. 4).

Effect estimates were larger for males (19%–34% increase during exposure years) than females (10%–19% increase) in primary healthcare (Supplementary Fig. 5); however, the absolute increase was similar between genders (Supplementary Fig. 6).

The increases in healthcare contacts observed in the original analyses did not show in the negative control analyses (see Supplementary Figs.7, 8, and 9). Negative control for eating disorder showed a similar, generally decreasing pattern, however with lower estimate values.

The two sensitivity analyses with removed autumn 2019 and half-year adjusted did not yield any major differences in results compared to the main results.

## Discussion

### Key results

We found an increase in days of GP office contacts of 14%–25% attributable to the policy. Overall GP office contacts and consultations for respiratory tract infections were considerably increased attributable to the school absence limit, but we found no apparent policy effect on consultations for mental health diagnoses. In specialist healthcare, we found weak or no effects on the overall use of somatic or specialist mental health services, we found a slight increase in contacts for ear–nose–throat diagnoses. Identified effects were mainly limited to upper secondary years.

### Strengths and limitations

Our results rely on the assumption of parallel age trends between the exposed and unexposed cohorts in the counterfactual absence of the school reform. We largely found support for the parallel trend assumption before the onset of exposure. However, this was less clear for specialist health services. Furthermore, if the effects of age are not linear, parallel trends before the first year of upper secondary might not ensure parallel trends over the entire age span. Therefore, only clear jumps in contacts/diagnoses also coinciding with exposure have been interpreted as possible absence-limit consequences. In addition, we utilized information from cohorts exposed at different ages.

Furthermore, our investigation of outcomes presumably unrelated to the school absence limit reform did not indicate strong confounding effects on these negative control measures.

Because of the COVID-19 pandemic, we chose to end follow-up at the end of 2019. Observation time after upper secondary was, therefore, limited, and should be interpreted with caution. However, sensitivity analyses showed that seasonal variation had limited effects on the results.

### Interpretation

In general practice, we found between 14% and 25% more contacts and 18%–30% more consultations among exposed cohorts. This corresponds well to the findings from a Norwegian cohort study [2], which found a 30% increase in consultations with GP and out-of-hours offices, and a 32% increase in telephone contact in the autumn of 2016, as well as to the evaluation report [3]. The effect seems to be somewhat smaller for third school year, which corresponds to the first year of apprenticeship for vocational programs, where different rules for sickness absence apply. Our findings of little evidence of any changes in the use of out-of-hours services were expected, as certificates are preferentially obtained at the GP offices rather than at out-of-hours services.

The effect on general practice use does not seem to spill over to more referrals to specialist healthcare in general, implying that excess health problems were taken care of in primary care and that the policy did not lead to improved detection of severe disease.

Our findings of a doubling of respiratory tract infections correspond well with the finding of an IRR of 2.21 by previous research [2]. As a remaining increase after the three exposed years was only found corresponding to autumn 2019, we found limited evidence supporting a prolonged trend after ended upper secondary school.

The absence limit was not followed by any substantial change in GP consultations for mental health. This is in contrast to previous evidence [2, 3]. However, these previous studies assessed a shorter time span after policy implementation. Although mental health complaints have increased in general practice [8], the trend does not seem to be caused by the absence policy.

Help-seeking behaviour did not seem to change beyond upper secondary, although we cannot exclude a possible effect for respiratory infections. However, limited follow-up after completion of upper secondary education limits the possibility of assessing whether the sickness absence limit led to lasting changes in help-seeking.

The slightly increased likelihood of receiving specialist care for ear–nose–throat diagnoses both during and after upper secondary corresponds well to the increase in respiratory tract infections in primary care. The time delay from the referral date to elective contact could explain the prolonged effect. This poses the question of whether there was an unmet need before the policy or possible inadequate gatekeeping in primary care. As contacts for eating disorders decrease during exposure years also in the negative control group, this shift is likely caused by something other than the absence policy, possibly due to changes in age trends over time.

In addition to the possibility of sick students feeling too stigmatized to visit or not believing a doctor could help [3], there is a possibility that the costs of certificates and consultations could represent a barrier for some students. Not gaining a certificate when needed may lead to attending school when sick or gaining too much-uncertified absence.

Our findings of stronger effects among children of higher-educated parents are not likely to reflect a higher need but could reflect an ability to pay. Previous studies have either found weak evidence of social inequalities in health among adolescents [17] or low socioeconomic status to be associated with poorer health and more use of GP services [18, 19]. From the age of 16, a consultation in a GP or out-of-hours office leads to an out-of-pocket payment [4], and additional fees for an absence certificate may also apply. However, absolute differences between educational groups were small.

As females see GPs more often than males from early teenage years [8], a similar absolute increase in contacts corresponds to a stronger relative effect estimate among males. The existing sex differences might thus be somewhat reduced because of the forced contact.

Our results confirm how the absence policy puts a strain on GP services. Further research is warranted to examine the adolescent health effects of this change in healthcare use.

### Generalizability

Our results may exemplify how a reform increasing adolescents’ use of GP services influences the healthcare use. Although the studied reform did put further strain on the GP system, the gatekeeper function seems to have mostly prevented an increased pressure on specialist care services.

### Conclusions

The school absence policy led to an increase in contact with GP offices, but not to the specialist healthcare. While the number of GP consultations for respiratory tract infections doubled, mental health diagnoses did not increase due to the policy. For diagnoses in specialist care, there was a slight increase in contacts for selected ear–nose–throat diagnoses. We found no major changes in help-seeking after upper secondary school years.

## Supplementary Material

cmae042_suppl_Supplementary_Material

## Data Availability

The data underlying this article were provided by third parties (Statistics Norway, the Norwegian Patient Registry, and the Control and Payment of Health Reimbursement Register) by permission and are not publicly available due to privacy legislations. Data may be requested from data owners upon ethical clearance.

## References

[1] The Norwegian Directorate for Education and Training. Fraværsgrense Udir-3-2016 [School absence limit Udir-3-2016]. 2020 ]; Retireved from https://www.udir.no/regelverkstolkninger/opplaring/Vitnemal/fravarsgrense---udir-3-2016/ (22 May 2024, date last accessed).

[2] Bakken IJ , Wensaas KA, Furu K, et al. Generalpractice consultations and use of prescription drugs after changes to school absence policy [Legesøkning og legemiddeluttak etter innføring av nye fraværsregler.]. Tidsskr Nor Laegeforen 2017;137:1178–1184. 10.4045/tidsskr.17.0427 28871761

[3] Drange N , Gjefsen H, Kindt MT, Rogstad J. Suksess og besvær, Evaluering av fraværsgrensen i videregående skole 2016-2019. Sluttrapport [Success and inconvenience, Evaluation af the school absence limit in upper secondary school 2016-2019. Final report]. Utdanningsdirektoratet, The Norwegian Directorate for Education and Training: Fafo, 2020 ISBN 978-82-324-0553-4 (Online version).

[4] Saunes IS , Karanikolos M, Sagan A. Norway: health system review. Health Syst Transit 2020;22:1–163. 32863241

[5] Forskrift om kommunens helsefremmende og forebyggende arbeid i helsestasjons- og skolehelsetjenesten [Regulations on municipalities’ health promoting and preventative work in the infant healthcare program and the school health service]. 2018.

[6] GPs and emergency primary health care [database on the Internet]. 2023 https://www.ssb.no/en/statbank/list/fastlegetj (11 December 2023, date last accessed).

[7] *Births and Deaths [Database on the Internet]*. 2023 https://www.ssb.no/en/befolkning/fodte-og-dode (19 December 2023, date last accessed).

[8] Wahlberg K , Pape K, Austad B, et al. Use of general practitioner services among youth and young adults in Norway from 2006 to 2021. Scand J Prim Health Care 2023;41:505–15. 37966167 10.1080/02813432.2023.2280045 PMC11001332

[9] Halfon N , Larson K, Lu M, et al. Lifecourse health development: past, present and future. . Matern Child Health J 2014;18:344–65. 10.1007/s10995-013-1346-2 23975451 PMC3890560

[10] Mauerhofer A , Berchtold A, Michaud PA, et al. GPs’ role in the detection of psychological problems of young people: a population-based study. Br J Gen Pract 2009;59:e308–14. 10.3399/bjgp09X454115 19761659 PMC2734378

[11] Huntington-Klein N. Chapter 18—difference-in-differences. 2023. *The Effect: An Introduction to Research Design and Causality [Internet]*. https://theeffectbook.net/ch-DifferenceinDifference.html?panelset=stata-code&panelset1=stata-code2&panelset2=stata-code3 (29 August 2024, date last accessed).

[12] Norwegian Agency for Quality Assurance in Education (NOKUT). *General information about education in Norway*. 2023 https://www.nokut.no/en/norwegian-education/general-information-about-education-in-norway/ (19 December 2023, date last accessed).

[13] Landsem MM , Magnussen J. The effect of copayments on the utilization of the GP service in Norway. Soc Sci Med 2018;205:99–106. 10.1016/j.socscimed.2018.03.034 29677584

[14] Olsen CB , Melberg HO. Did adolescents in Norway respond to the elimination of copayments for general practitioner services?. Health Econ 2018;27:1120–30. 10.1002/hec.3660 29663571

[15] Correia S , Guimarães P, Zylkin T. Fast Poisson estimation with high-dimensional fixed effects. The Stata J 2020;20:95–115. 10.1177/1536867x20909691

[16] Lipsitch M , Tchetgen Tchetgen E, Cohen T. Negative controls: a tool for detecting confounding and bias in observational studies. Epidemiology 2010;21:383–8. 10.1097/EDE.0b013e3181d61eeb 20335814 PMC3053408

[17] Joffer J , Flacking R, Bergström E, et al. Self-rated health, subjective social status in school and socioeconomic status in adolescents: a cross-sectional study. BMC Public Health. 2019;19:785. 31221114 10.1186/s12889-019-7140-3 PMC6587278

[18] Mosquera PA , Waenerlund AK, Goicolea I, et al. Equitable health services for the young? A decomposition of income-related inequalities in young adults’ utilization of health care in Northern Sweden. Int J Equity Health 2017;16:20. 28100232 10.1186/s12939-017-0520-3 PMC5241958

[19] Reiss F. Socioeconomic inequalities and mental health problems in children and adolescents: a systematic review. Soc Sci Med 2013;90:24–31. 10.1016/j.socscimed.2013.04.026 23746605

