# Supplementary material for: School absence policy and healthcare use: a difference-in-difference cohort analysis
Source: Fam Pract. 2024 Sep 6;42(2):cmae042. doi: 10.1093/fampra/cmae042 (PMC11879198; doi:10.1093/fampra/cmae042)
Supplement: cmae042_suppl_Supplementary_Material [file cmae042_suppl_supplementary_material.pdf]

Supplementary material

Additional method description

Outcomes:

We defined consultations as contacts where reimbursement codes 2ad, 2ae, 2ak, 2ed, 2fk, 2af, 11ad, 11ak were used. E-consultations (code 2ae) were introduced in 2013 and are a less homogeneous group than physical consultations. However, they were infrequently used during the observed study period. When counting days of contact we additionally included codes 1ad, 1ak, 1bd, 1bk, 1be, 1e, 1g, 1h and 1i.

Covariates:

Immigration status was divided into 6 categories, based on the person and their parents' countries of birth. As a measure of parental socioeconomic status, we used the highest level of parental education when the person was 16 years old (or at current age for those under 16). Parental education had four categories: long (>4 years) and short higher education, upper secondary schooling and primary school. We further dichotomized it as higher education or lower. We defined an indicator variable identifying all participants living in the same municipality per year, to account for differences between municipalities and years.

Supplementary table 1

Selected diagnosis groups and codes used for coding from ICPC-2 or ICD-10.

**Diagnosis group**	**ICPC-2 codes**
Respiratory tract infections	R05, R09-R23, R71-R83, H71-H74
Mental health diagnoses	P01-P26, P28, P29, P70-P99
Acne	S96
Nevus	S82
**Diagnosis group**	**ICD-10 codes**
Affective disorder	F30-F39
Ear-nose-throat	J30-J36
Eating disorder	F50
Fracture	S52, S62

Supplementary table 2

Percentage of school year group enrolled in school that would be affected by the absence limit if it was an exposure year (i.e. general studies and vocational studies before the apprenticeship period).

	Schoolyear				
**Exposure group**	1st	2nd	3rd	1st after	2nd after
0	94.16	91.22	73.60	13.52	7.45
1	93.99	91.21	73.68	12.78	7.45
2	93.75	90.68	73.38	12.54	7.52
3	94.11	92.48	73.81	12.04	

Supplementary figure 1

Estimated incidence rate ratio (IRR) of consultations at GP offices from a difference-in-difference model. By school year and exposure groups, estimates reflecting the change attributable to the school absence policy. School years were defined from July 1st to June 30th the following year and named by the corresponding grade name. Exposure groups are defined by expected age at start of exposure.

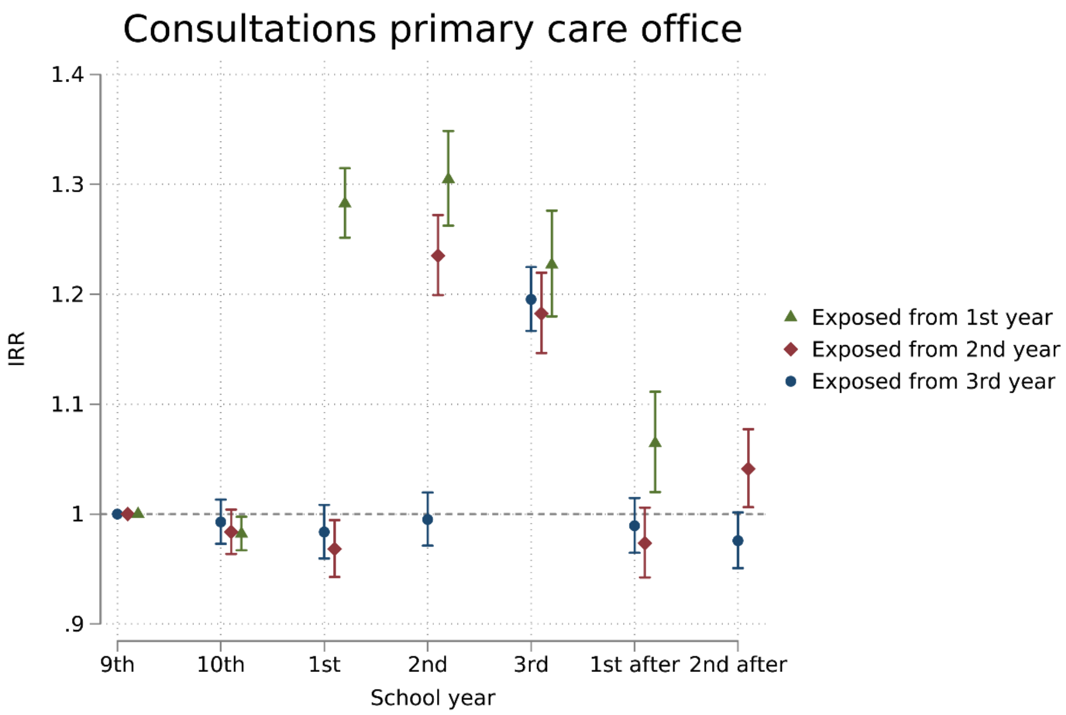

Supplementary figure 2

Estimated incidence rate ratio (IRR) of consultations at OOH services from a difference-in-difference model. By school year and exposure groups, estimates reflecting the change attributable to the school absence policy. School years were defined from July 1st to June 30th the following year and named by the corresponding grade name. Exposure groups are defined by expected age at start of exposure.

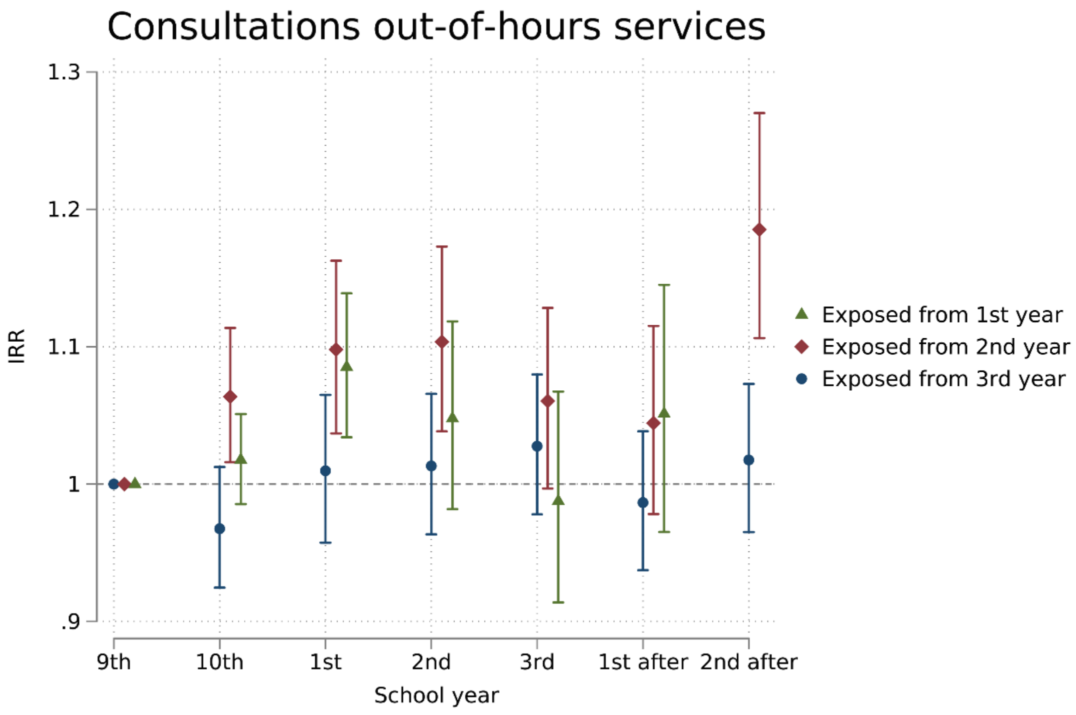

Supplementary figure 3

Estimated incidence rate ratio (IRR) of contact types (a-c) from a difference-in-difference model. By school year, exposure groups and parental education, estimates reflecting the change attributable to the school absence policy. School years were defined from July 1st to June 30th the following year and named by the corresponding grade name. Exposure groups are defined by expected age at start of exposure.

a) Days of GP office contacts

b) Probability of somatic specialist health care contact

c) Probability of specialist mental health care contact

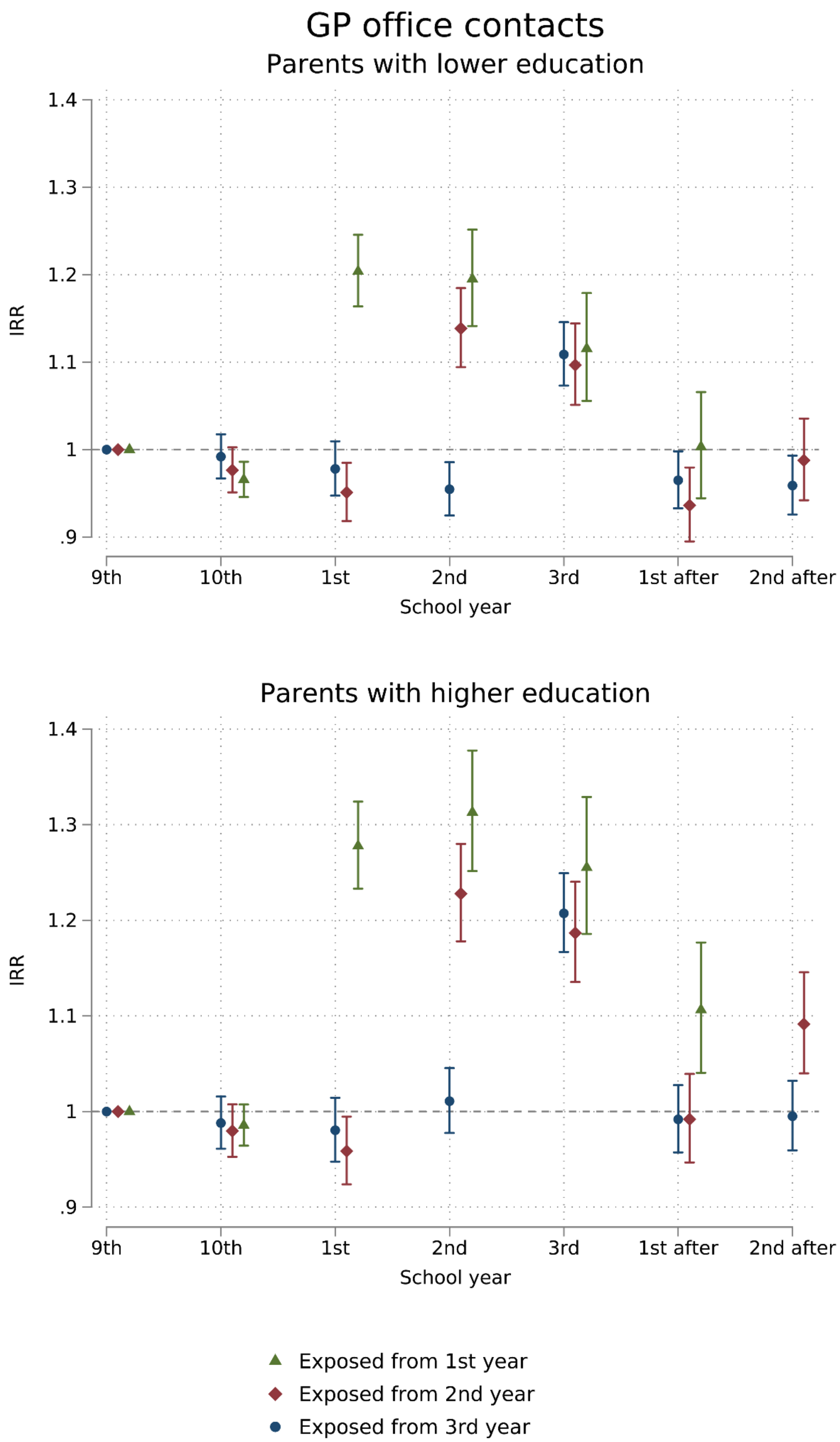

GP office contacts
Parents with lower education
IRR
1.4
1.3
1.2
1.1
1
.9
9th
10th
1st
2nd
3rd
1st after
2nd after
School year
Parents with higher education
IRR
1.4
1.3
1.2
1.1
1
.9
9th
10th
1st
2nd
3rd
1st after
2nd after
School year
Exposed from 1st year
Exposed from 2nd year
Exposed from 3rd year

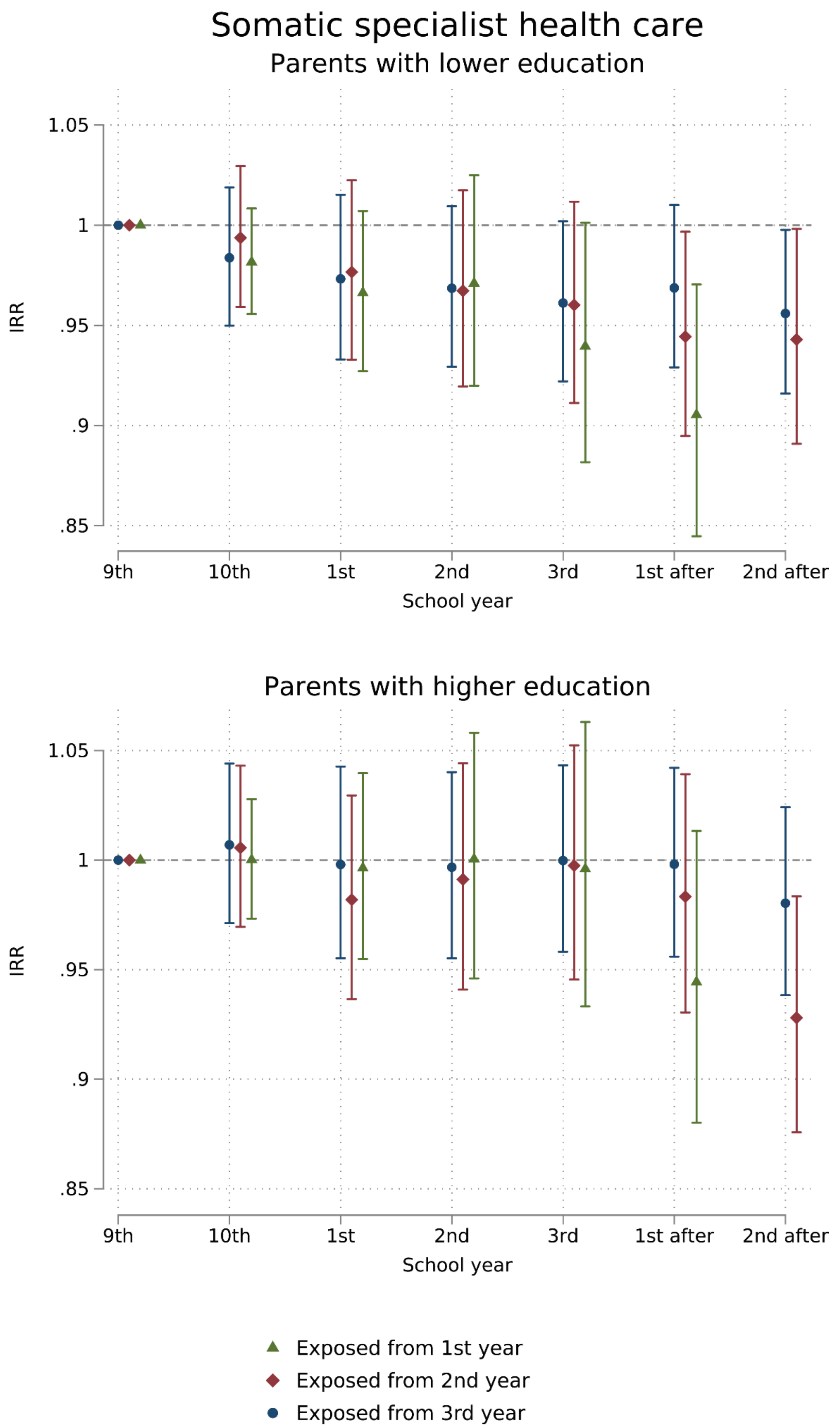

Somatic specialist health care
Parents with lower education
IRR
1.05
1
.95
.9
.85
9th
10th
1st
2nd
3rd
1st after
2nd after
School year
Parents with higher education
IRR
1.05
1
.95
.9
.85
9th
10th
1st
2nd
3rd
1st after
2nd after
School year
Exposed from 1st year
Exposed from 2nd year
Exposed from 3rd year

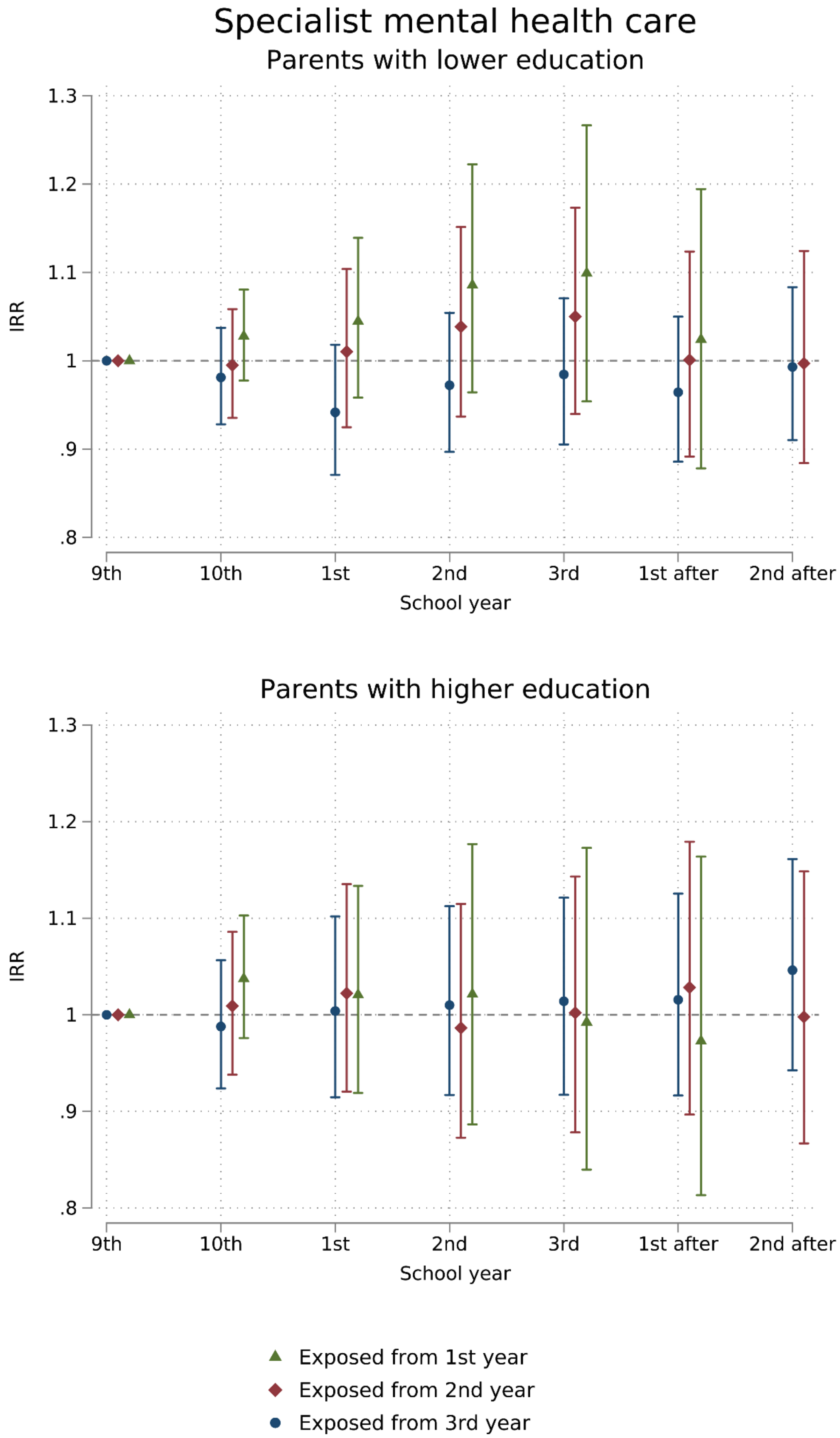

Specialist mental health care
Parents with lower education
IRR
1.3
1.2
1.1
1
.9
.8
9th
10th
1st
2nd
3rd
1st after
2nd after
School year
Parents with higher education
IRR
1.3
1.2
1.1
1
.9
.8
9th
10th
1st
2nd
3rd
1st after
2nd after
School year
Exposed from 1st year
Exposed from 2nd year
Exposed from 3rd year

Supplementary figure 4

Estimated increase in number of days of GP office contacts from a difference-in-difference model. By school year, exposure groups and highest parental education, estimates reflecting the change attributable to the school absence policy. School years were defined from July 1st to June 30th the following year and named by the corresponding grade name. Exposure groups are defined by expected age at start of exposure.

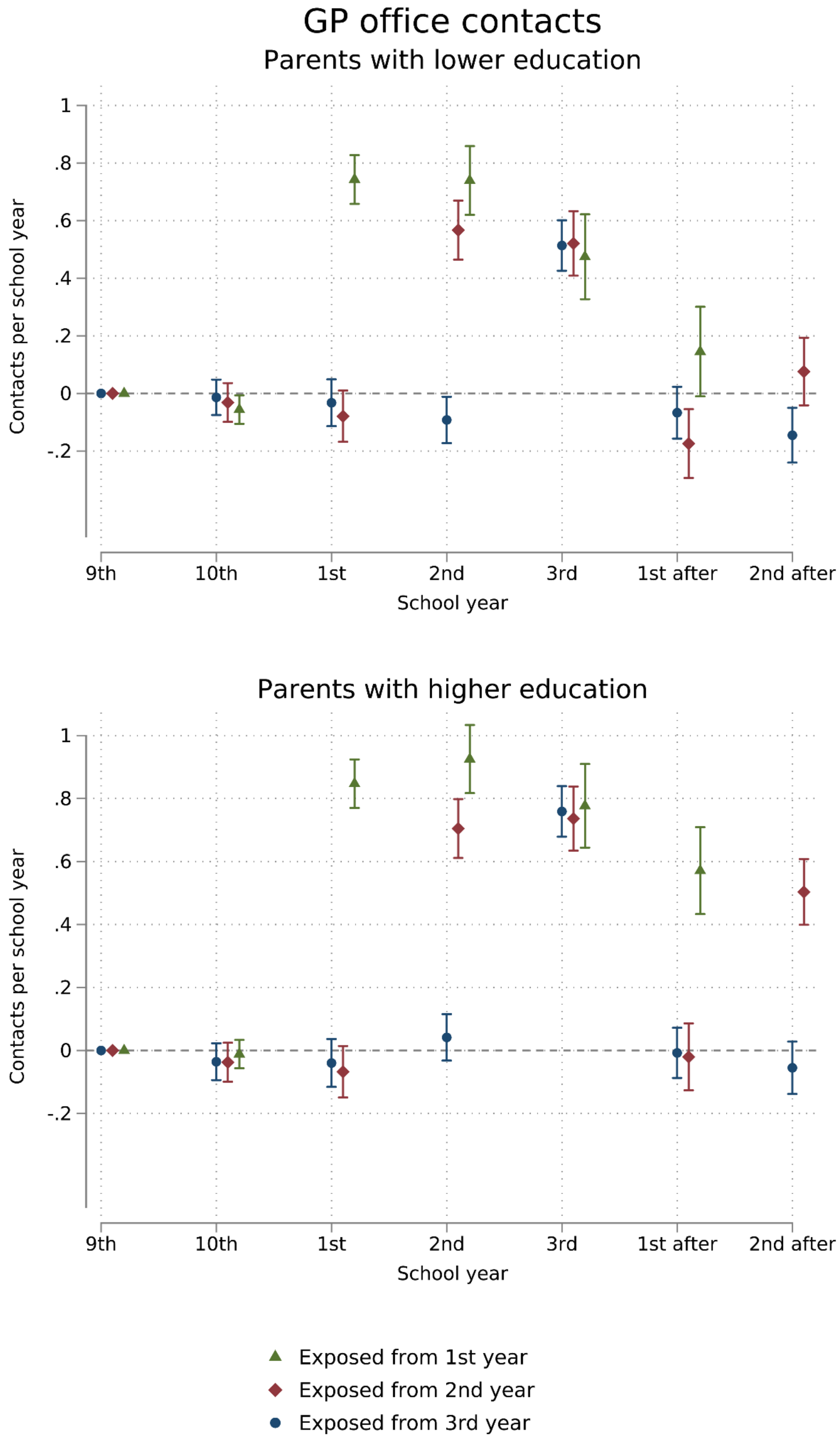

GP office contacts
Parents with lower education
Contacts per school year
1
.8
.6
.4
.2
0
-.2
9th
10th
1st
2nd
3rd
1st after
2nd after
School year
Parents with higher education
Contacts per school year
1
.8
.6
.4
.2
0
-.2
9th
10th
1st
2nd
3rd
1st after
2nd after
School year
Exposed from 1st year
Exposed from 2nd year
Exposed from 3rd year

Supplementary figure 5

Estimated incidence rate ratio (IRR) of contact types (a-c) from a difference-in-difference model. By school age, exposure groups and sex, estimates reflecting the change attributable to the school absence policy. School years were defined from July 1st to June 30th the following year and named by the corresponding grade name. Exposure groups are defined by expected age at start of exposure.

a) Days of GP office contacts
b) Probability of somatic specialist health care contact
c) Probability of specialist mental health care contact

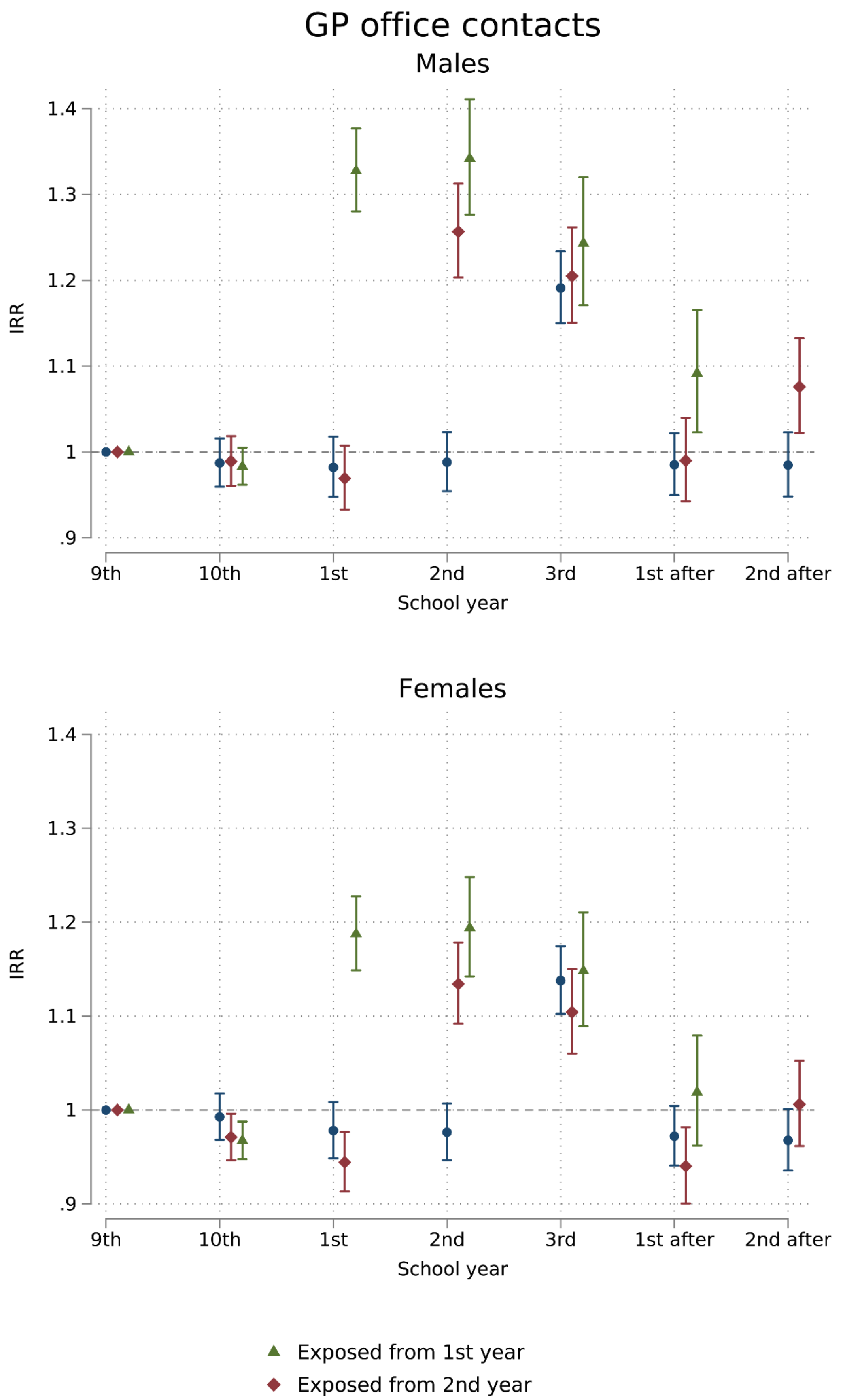
GP office contacts
Males
1.4
1.3
1.2
1.1
1
.9
IRR
9th
10th
1st
2nd
3rd
1st after
2nd after
School year
Females
1.4
1.3
1.2
1.1
1
.9
IRR
9th
10th
1st
2nd
3rd
1st after
2nd after
School year
Exposed from 1st year
Exposed from 2nd year
Exposed from 3rd year

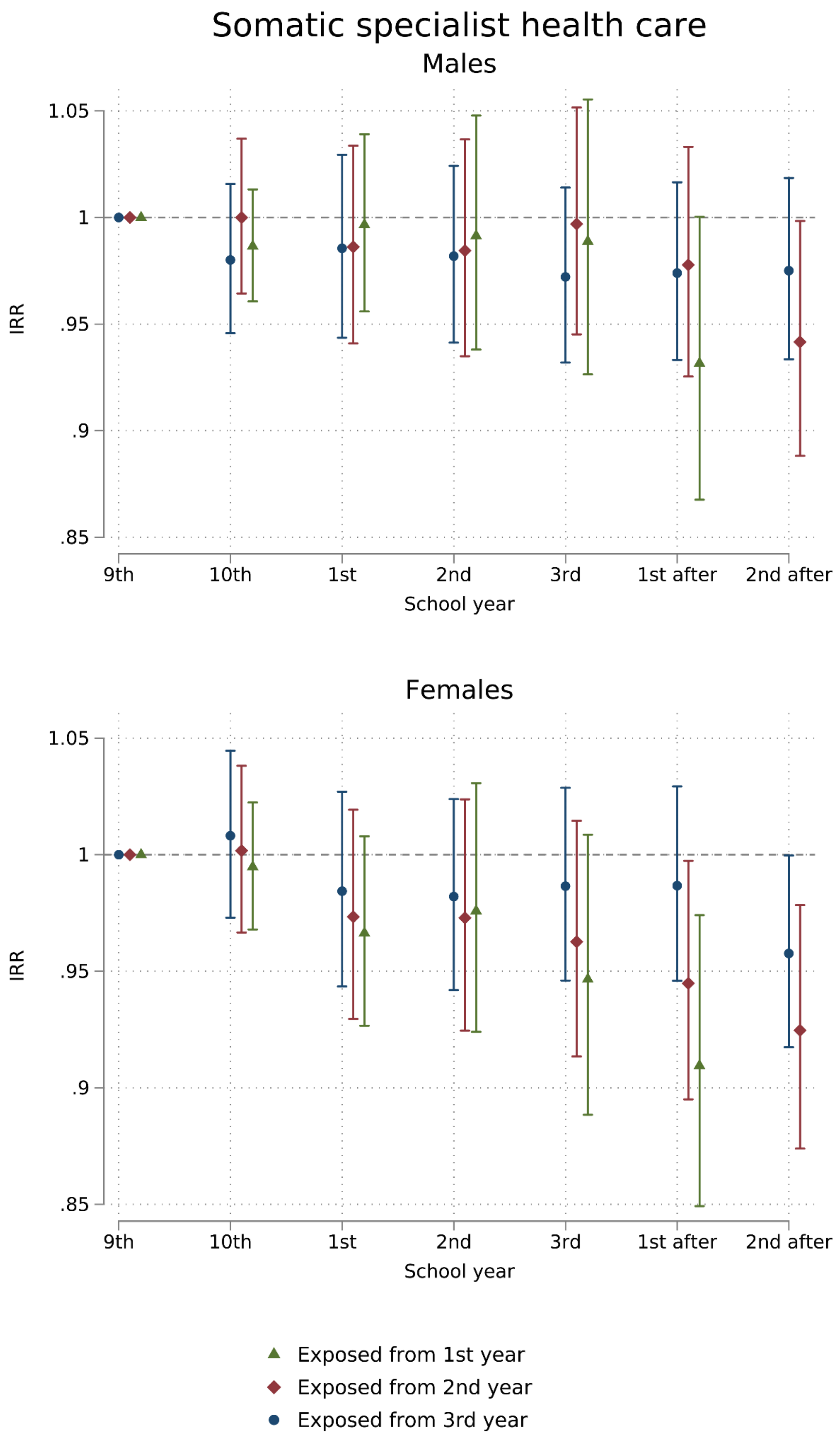

Somatic specialist health care
Males
1.05
1
.95
.9
.85
IRR
9th
10th
1st
2nd
3rd
1st after
2nd after
School year
Females
1.05
1
.95
.9
.85
IRR
9th
10th
1st
2nd
3rd
1st after
2nd after
School year
Exposed from 1st year
Exposed from 2nd year
Exposed from 3rd year

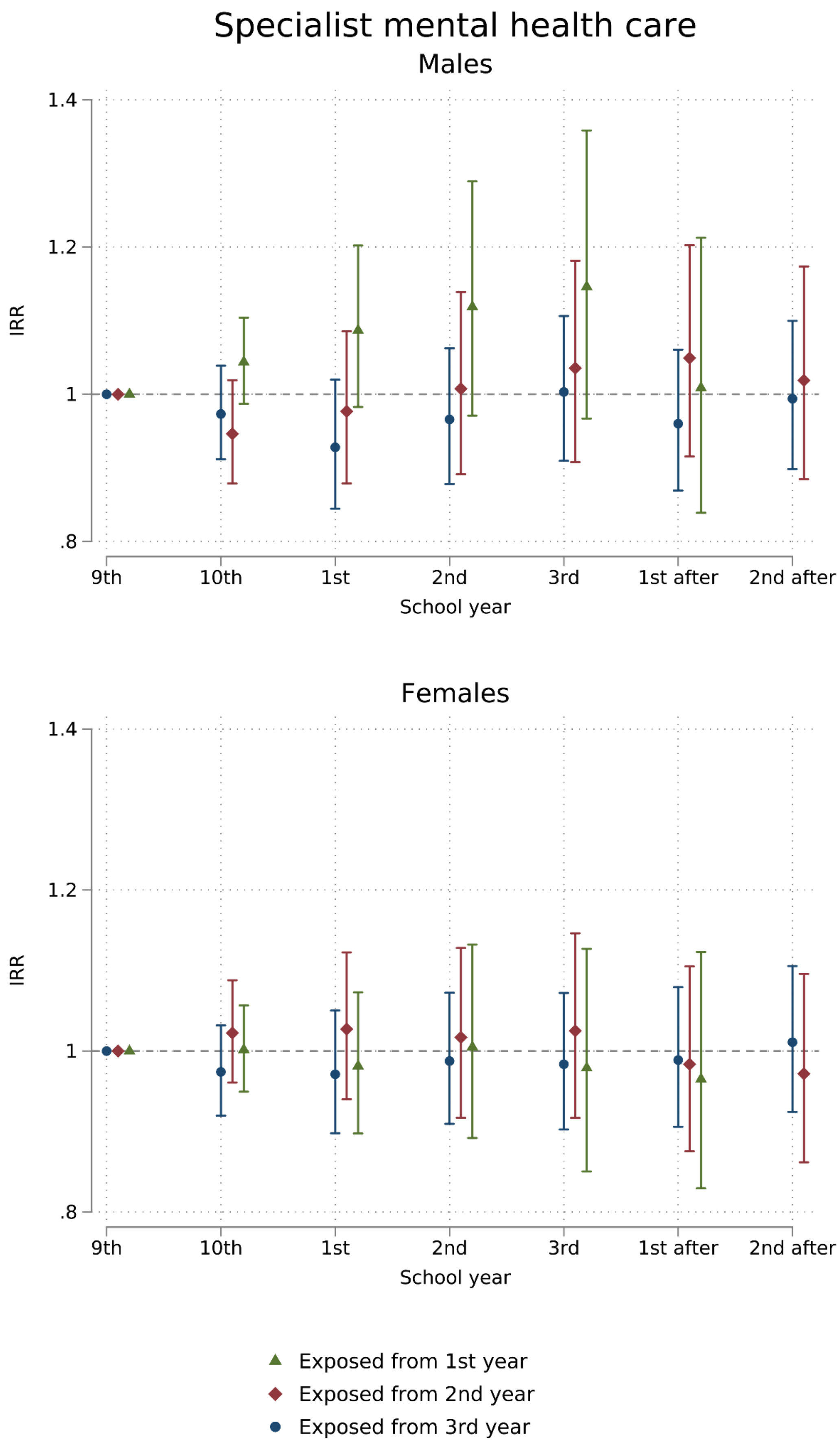

Specialist mental health care
Males
1.4
1.2
1
.8
IRR
9th
10th
1st
2nd
3rd
1st after
2nd after
School year
Females
1.4
1.2
1
.8
IRR
9th
10th
1st
2nd
3rd
1st after
2nd after
School year
Exposed from 1st year
Exposed from 2nd year
Exposed from 3rd year

Supplementary figure 6

Estimated increase in number of days of GP office contacts from a difference-in-difference model. By school age, exposure groups and sex, estimates reflecting the change attributable to the school absence policy. School years were defined from July 1st to June 30th the following year and named by the corresponding grade name. Exposure groups are defined by expected age at start of exposure.

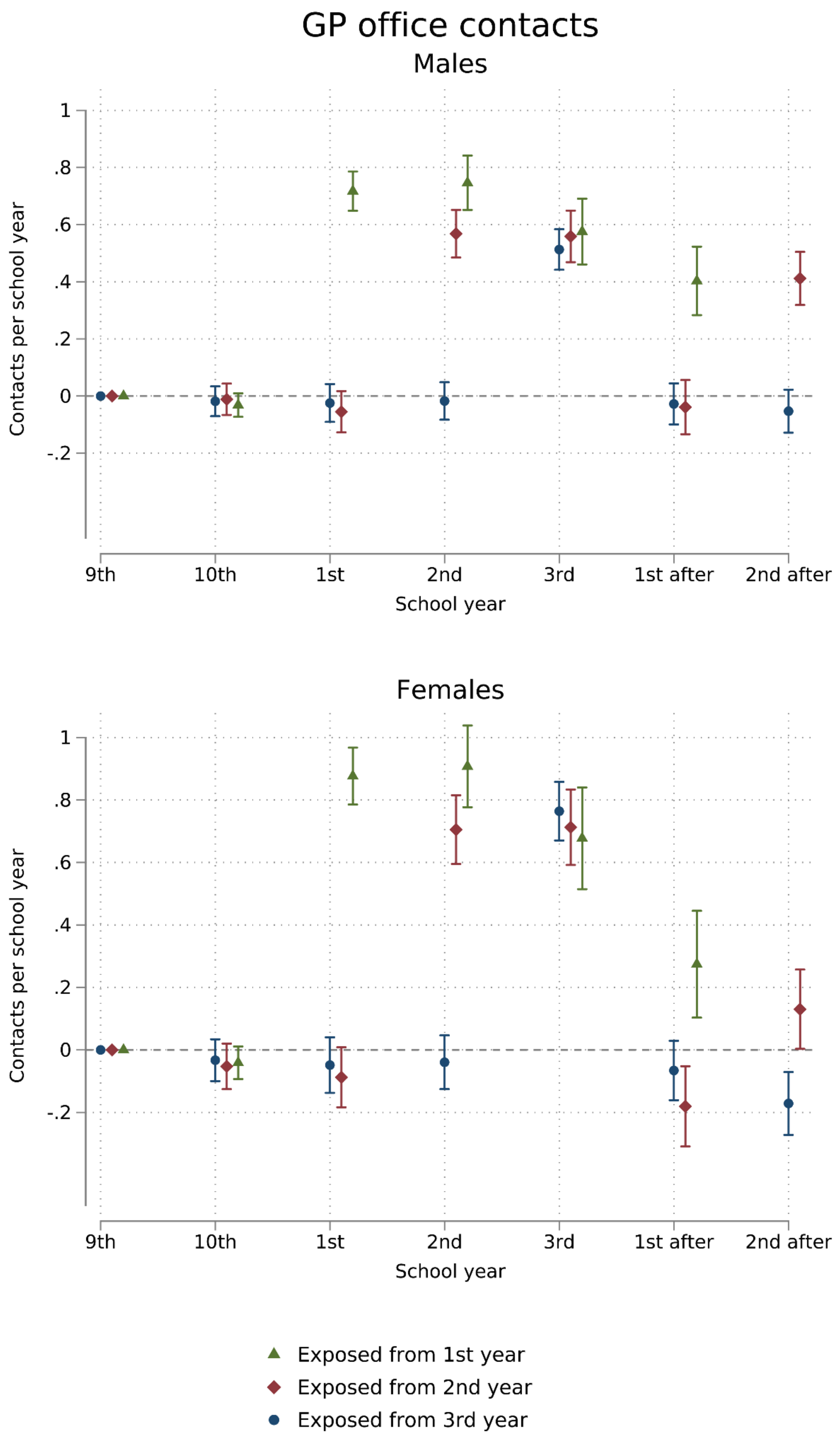

GP office contacts
Males
Contacts per school year
1
.8
.6
.4
.2
0
-.2
9th
10th
1st
2nd
3rd
1st after
2nd after
School year
Females
Contacts per school year
1
.8
.6
.4
.2
0
-.2
9th
10th
1st
2nd
3rd
1st after
2nd after
School year
Exposed from 1st year
Exposed from 2nd year
Exposed from 3rd year

Supplementary figure 7

Estimated incidence rate ratio (IRR) of contact types (a-c) for youth who have not started upper secondary school ("negative control") from a difference-in-difference model. By school age and exposure groups, estimates reflecting the change attributable to the school absence policy. School years were defined from July 1st to June 30th the following year and named by the corresponding grade name. Exposure groups are defined by expected age at start of exposure.

a) Probability of somatic specialist health care contact

b) Probability of specialist mental health care contact

c) Days of GP office contacts

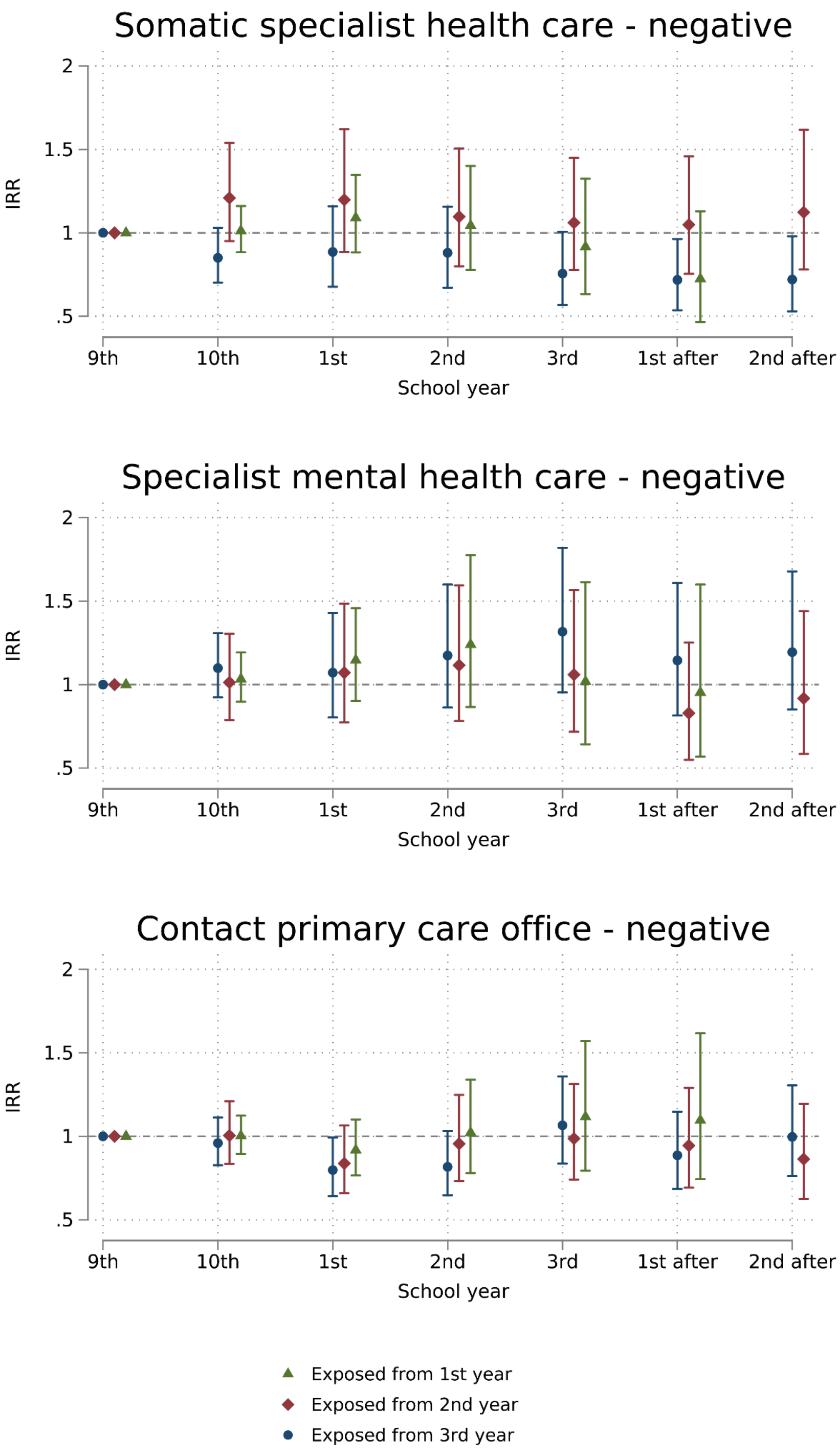
Somatic specialist health care - negative
IRR
2
1.5
1
.5
9th
10th
1st
2nd
3rd
1st after
2nd after
School year
Specialist mental health care - negative
IRR
2
1.5
1
.5
9th
10th
1st
2nd
3rd
1st after
2nd after
School year
Contact primary care office - negative
IRR
2
1.5
1
.5
9th
10th
1st
2nd
3rd
1st after
2nd after
School year
Exposed from 1st year
Exposed from 2nd year
Exposed from 3rd year

Supplementary figure 8

Estimated incidence rate ratio (IRR) of number of consultations with selected diagnoses in a GP office per school year for youth who have not started upper secondary school ("negative control") from a difference-in-difference model. By school year and exposure groups, estimates reflecting the change attributable to the school absence policy. School years were defined from July 1st to June 30th the following year and named by the corresponding grade name. Exposure groups are defined by expected age at start of exposure.

Estimates for nevus for those exposed from first year were omitted third year, and for those exposed from 2nd year were omitted 2nd year after upper secondary school due to collinearity.

Upper confidence limits over 45 in the nevus graph were set to 45 for readability.

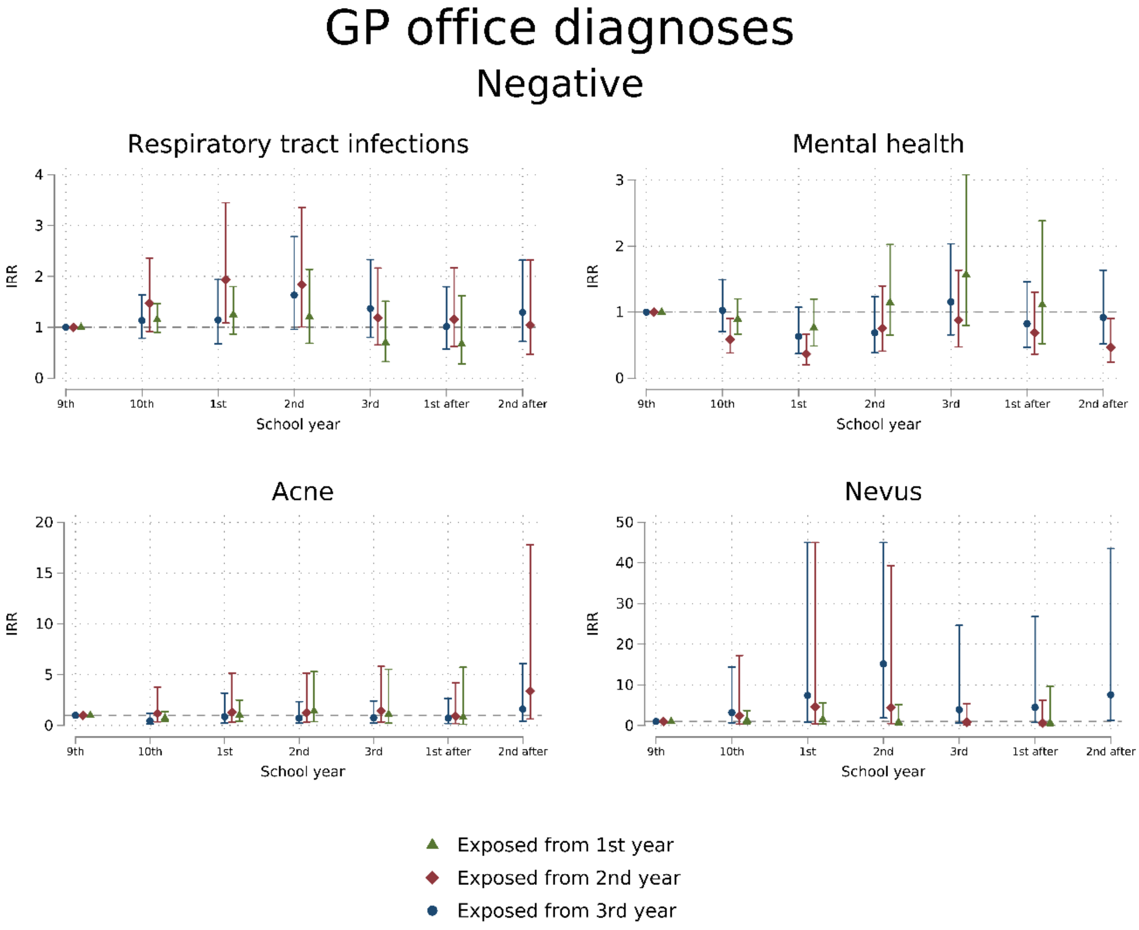

Supplementary figure 9

Estimated incidence rate ratio (IRR) of probability of contact with selected diagnoses in specialist health care per school year for youth who have not started upper secondary school ("negative control") from a difference-in-difference model. By school year and exposure groups, estimates reflecting the change attributable to the school absence policy. School years were defined from July 1st to June 30th the following year and named by the corresponding grade name. Exposure groups are defined by expected age at start of exposure.

Estimate for eating disorder for those exposed from 2nd year were omitted third year after upper secondary school due to collinearity.

Estimate, lower and/or upper confidence limits over 45 in the fracture graph were dropped or set to 45 for readability.

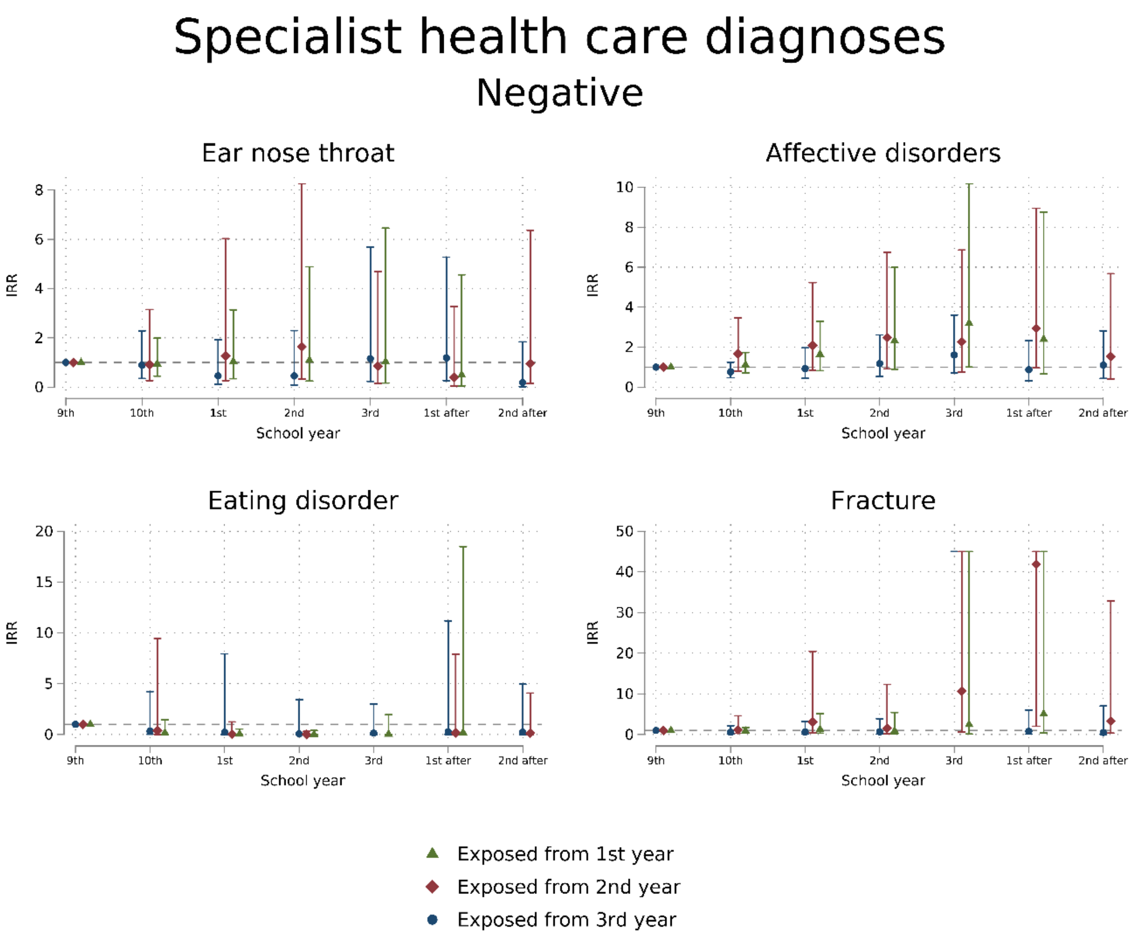
Specialist health care diagnoses
Negative
Ear nose throat
Affective disorders
Eating disorder
Fracture
IRR
School year
9th
10th
1st
2nd
3rd
1st after
2nd after
Exposed from 1st year
Exposed from 2nd year
Exposed from 3rd year
